# Exploring high-density corticomuscular networks after stroke to enable a hybrid Brain-Computer Interface for hand motor rehabilitation

**DOI:** 10.1186/s12984-023-01127-6

**Published:** 2023-01-14

**Authors:** Floriana Pichiorri, Jlenia Toppi, Valeria de Seta, Emma Colamarino, Marcella Masciullo, Federica Tamburella, Matteo Lorusso, Febo Cincotti, Donatella Mattia

**Affiliations:** 1 Neuroelectrical Imaging and Brain Computer Interface Lab, IRCCS Fondazione Santa Lucia, Via Ardeatina, 306, 00179 Rome, Italy; 2 Dept. of Computer, Control and Management Engineering, Sapienza University of Rome, Rome, Italy; 3 Neurology and Neurovascular Treatment Unit, Belcolle Hospital, Viterbo, Italy; 4 Laboratory of Robotic Neurorehabilitation (NeuroRobot Lab), Neurorehabilitation 1 Department, IRCCS Fondazione Santa Lucia, Rome, Italy

**Keywords:** Brain computer interface, EEG, EMG, Corticomuscolar coherence, Stroke, Graph theory, Neurorehabilitation, Upper limb, Hand motor task

## Abstract

**Background:**

Brain-Computer Interfaces (BCI) promote upper limb recovery in stroke patients reinforcing motor related brain activity (from electroencephalogaphy, EEG). Hybrid BCIs which include peripheral signals (electromyography, EMG) as control features could be employed to monitor post-stroke motor abnormalities. To ground the use of corticomuscular coherence (CMC) as a hybrid feature for a rehabilitative BCI, we analyzed high-density CMC networks (derived from multiple EEG and EMG channels) and their relation with upper limb motor deficit by comparing data from stroke patients with healthy participants during simple hand tasks.

**Methods:**

EEG (61 sensors) and EMG (8 muscles per arm) were simultaneously recorded from 12 stroke (EXP) and 12 healthy participants (CTRL) during simple hand movements performed with right/left (CTRL) and unaffected/affected hand (EXP, UH/AH). CMC networks were estimated for each movement and their properties were analyzed by means of indices derived ad-hoc from graph theory and compared among groups.

**Results:**

Between-group analysis showed that CMC weight of the whole brain network was significantly reduced in patients during AH movements. The network density was increased especially for those connections entailing bilateral non-target muscles. Such reduced muscle-specificity observed in patients was confirmed by muscle degree index (connections per muscle) which indicated a connections’ distribution among non-target and contralateral muscles and revealed a higher involvement of proximal muscles in patients. CMC network properties correlated with upper-limb motor impairment as assessed by Fugl-Meyer Assessment and Manual Muscle Test in patients.

**Conclusions:**

High-density CMC networks can capture motor abnormalities in stroke patients during simple hand movements. Correlations with upper limb motor impairment support their use in a BCI-based rehabilitative approach.

**Supplementary Information:**

The online version contains supplementary material available at 10.1186/s12984-023-01127-6.

## Background

Disability after stroke is largely due to residual upper limb motor deficit [1]. This latter is the target of several novel rehabilitation approaches including those based on Brain-Computer Interfaces—BCIs [2, 3]. BCIs for post-stroke motor rehabilitation rely on the principle that reinforcement of close-to-normal motor related brain activity (most frequently derived from electroencephalogram—EEG), results in an improvement of motor function [4]. Hybrid BCIs exploit physiological signals other than brain activity, such as muscular activity derived from surface electromyography (EMG), in order to increase the classification performance [5]. However, such hybrid systems could be employed in the context of post-stroke motor rehabilitation to monitor motor abnormalities, such as spasticity, co-contractions, motor overflow [6–10] in order to reinforce close-to-normal muscular activation.

To this purpose, we recently explored the potential of cortico-muscular coherence (CMC) patterns derived from high-density EEG/EMG as a feature for a rehabilitative hybrid BCI in healthy subjects performing simple hand movements (most commonly employed in BCI paradigms) obtaining high classification performances with the most discriminant EEG–EMG features [11]. With respect to currently available hybrid BCI systems which combine different signals at the classification stage, CMC can be conceived as an intrinsically hybrid feature per se allowing simultaneous monitoring of the interaction between brain (EEG) and muscular (EMG) activity.

Indeed, CMC is a measure of brain-muscle interplay during movement, derived from EEG–EMG coupling within motor relevant EEG frequency bands [12]. CMC is altered after stroke, showing mainly a reduction in EEG–EMG coupling [13]. Until recently, most CMC studies in stroke patients have limited the observation to few EEG electrodes in the affected hemisphere and the target muscle [12–15]. Similarly, the implementation of CMC-based BCIs has been limited to few EEG–EMG couples determined a priori [16]. However, the complexity of post-stroke recovery is such that several brain regions and muscles participate in post-lesional re-arrangements [17–20]. Lately, stroke-related CMC studies have broadened the observation to multi-channel recordings to describe complex phenomena such as the contralesional hemisphere contribution [21] or the abnormal recruitment of antagonists and proximal muscles [14, 22, 23]. All this evidence supports the potential role of CMC control feature in a rehabilitative BCI paradigm for its capability to encode both volitional control over movement and possible deviations from the physiological motor system activation, thus well beyond the purpose of increasing system classification performance.

A successful introduction of CMC control feature in rehabilitative BCIs requires to first identify which properties of the widespread corticomuscular network (namely which EEG–EMG features) would best outline the complexity of post-stroke motor deficit to ensure that such hybrid BCI will favor functional motor recovery and eventually discourage maladaptive changes.

In the present study, CMC patterns were estimated by means of high-density recordings to best capture the widespread corticomuscular network properties in stroke patients during the execution of simple hand movements such as grasping and finger extension. With this aim, the network's properties were then characterized by means of ad hoc indices derived from a graph theoretical approach [24]. Statistical analysis was performed to outline differences between healthy subjects and patients, performing the movements both with the affected and unaffected hand (AH, UH), and to seek correlation with upper limb motor impairment as assessed by clinical scales.

## Methods

### Participants

Twelve stroke participants were included in the study (EXP group: 6 females/6males age 52.5 ± 18.5 yr) according to the following inclusion criteria: (1) first-ever unilateral, cortical, subcortical, or mixed stroke, caused by ischemia or hemorrhage (confirmed by magnetic resonance imaging), that occurred 3 to 12 months prior to study inclusion; (2) upper limb hemiparesis that was caused by the stroke; and (3) age between 18 and 80 years. The exclusion criteria were the presence of: (i) neuropsychological deficits preventing the ability to understand the instructions related to the experiment; (ii) concomitant diseases affecting the upper limb motor function (i.e., orthopedic injuries or other neurologic diseases affecting reaching or grasping); (iii) spasticity of each segment of the upper limb scored higher than 4 on the Modified Ashworth Scale (MAS [25]). All stroke participants were recruited within the inpatients and outpatients services of Fondazione Santa Lucia, IRCCS, Rome, Italy and were undergoing a rehabilitative treatment (usual care).

Twelve healthy participants (CTRL group: 9 females/3 males, age 43.6 ± 15.3 yr) participated in the study as a control group. Subjects did not present any evidence/known history of neurologic or neuromuscular disorders, nor any permanent/transient condition that could affect upper limb motor function.

The study was approved by the local ethics board at Fondazione Santa Lucia, IRCCS, Rome, Italy (CE PROG.752/2019) and all the participants signed an informed consent.

Clinical and functional evaluation was performed by expert physiotherapists before data acquisition (same day) by means of the following scales: (i) the National Institute of Health Stroke Scale (NIHSS) to assess general impairment derived from stroke [26]; (ii) the Manual Muscle Test (MMT) to assess strength in the paretic upper limb testing shoulder abduction, elbow flexion/extension and wrist flexion/extension [27]; (iii) the MAS scale to assess spasticity of shoulder, elbow and wrist muscles [25]. The upper extremity section of the Fugl-Meyer Assessment scale (FMA), comprising the four sub-scales “Upper Limb”, “Wrist”, “Hand”, “Coordination and Velocity” was performed to extensively describe the paretic upper limb residual function [28]. Handedness was assessed in all participants by means of the short form of the Edinburgh Handedness Inventory (EHI [29]).

### Experimental design and data acquisition

The EEG and EMG signals were acquired simultaneously and sampled at 1 and 2 kHz, respectively. 61-channel EEG was recorded from the scalp by means of active electrodes (Brain Products GmbH, Germany) arranged according to an extension of 10–20 International System (reference on left mastoid and ground on right mastoid). Surface bipolar EMG signals were recorded by means of Pico EMG sensors (Cometa S.r.l., Italy) from the following 16 muscles: extensor digitorum (ED), flexor digitorum superficialis (FD), lateral head of the triceps muscle (TRI), long head of the biceps brachii muscle (BIC), pectoralis major (PEC), lateral deltoid (Lat_DELT), anterior deltoid (Ant_DELT) and upper trapezius (TRAP) of both sides (L: left, R: right for healthy subjects, AH: affected hand, UH: unaffected hand for stroke participants). EEG and EMG signals were amplified by means of BrainAmp (Brain Products GmbH, Germany) and Wave plus 16 channels (Cometa S.r.l., Italy) amplifiers, respectively.

**Fig. 1:**
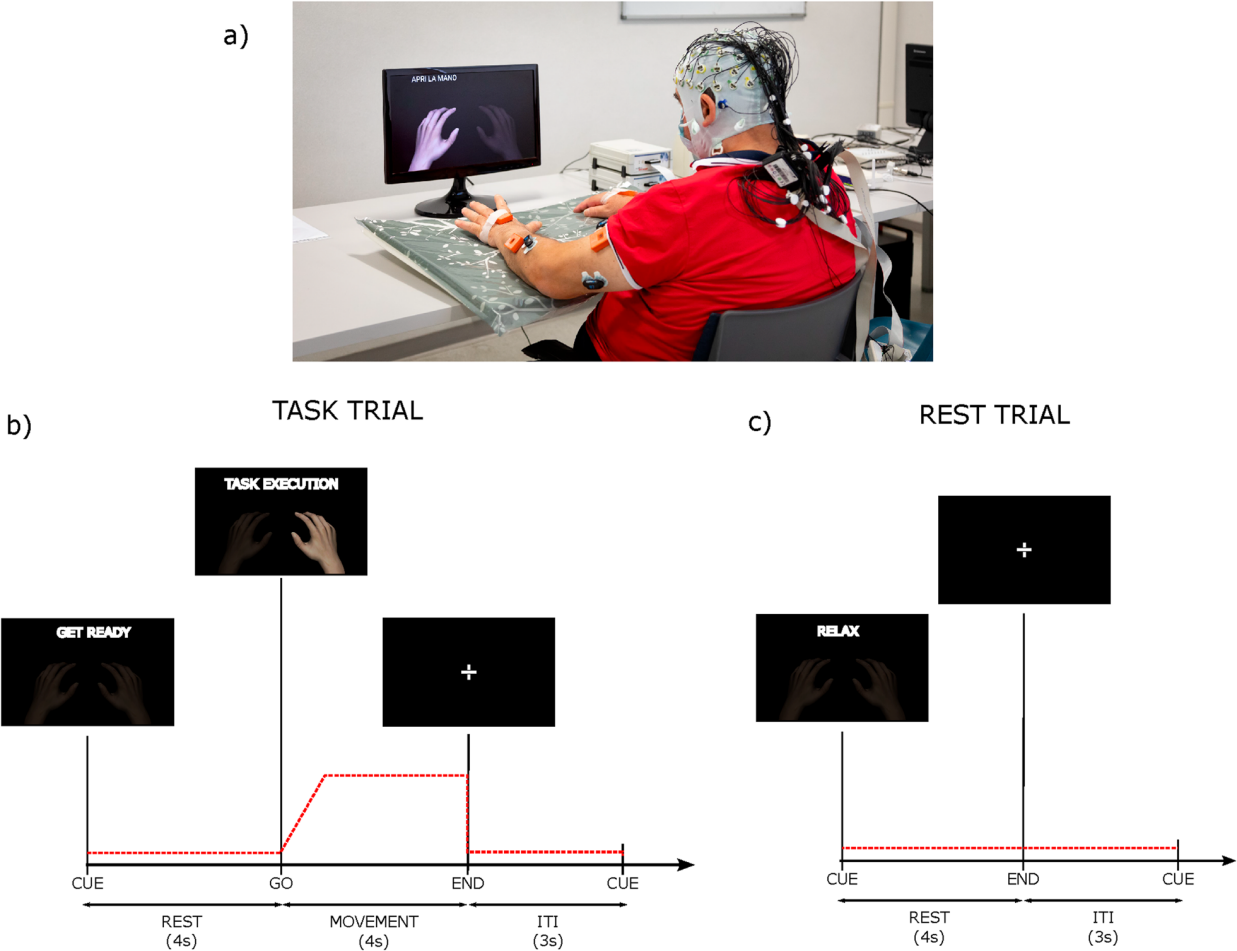
Experimental setting. **a** Participant setting; **b**, **c** experiment timeline for Task (movement) and rest trials. The red dotted line represents the activation profile required to correctly complete the task as for the target muscle (ED for Ext and FD for Grasp) and Rest. In addition to EEG and EMG data, the recording included also kinematic data. They were collected at 100 Hz by means of 8 IMUs (MTw Awinda, Xsens Technologies, The Netherlands). The IMUs were placed by a double-sided medical tape on the following anatomical points: hand, mid forearm, mid arm of both upper limbs, over the clavicular notch and at the lumbar vertebrae level. Such data were not included in this study

The experimental setting is illustrated in Fig. 1. All participants were seated in a comfortable chair or wheelchair if needed, with their forearms resting on a pillow placed over a table (Fig. 1a). Participants were presented with visual cues displayed on a screen (1 m distance). The experimental session consisted of 4 runs (intermingled with breaks adapted to the patients’ necessities) during which the participant was asked to perform finger extension (Ext) and grasping (Grasp) with the right and the left hand separately (UH, AH for stroke participants). Each run comprised 40 trials (20 “task” trials of 8 s each and 20 “rest” trials of 4 s each in random order). The inter-trial-interval lasted 3 s during which participants were required to fixate a cross in the middle of the screen. “Task” trials started with 4 s of preparation (”get ready” instruction) afterward a go stimulus appeared (”task” instruction) and the participant had to perform the task for 4 s (Fig. 1b). In “rest” trials participants had to relax for 4 s (“relax” instruction—Fig. 1c). Participants were instructed to perform the task as fast as they could and to hold it at 15% of Maximum Voluntary Contraction (MVC) of the target muscle until the end of the trial (the experimenter guided the participants via online visualization of EMG traces). MVCs were recorded for each muscle at the beginning of the experiment for 5 s. Stroke participants attempted the movements with their affected limb to the best of their own residual ability, following the same instructions.

### Data analysis

#### EEG–EMG data pre-processing

EEG data were band-pass filtered [3–60] Hz and Independent Component Analysis was used to remove ocular artifacts (Vision Analyzer 1.05 software, Brain Products GmbH, Gilching, Germany). EMG signals were downsampled to 1000 Hz, band-pass filtered [3–500] Hz and the electrocardiographic (ECG) component was rejected through template matching approach. A notch filter at 50 Hz was applied to remove power-line artifacts on both EEG and EMG signals. Task trials were segmented in 8 s epochs while Rest trials were segmented in 4 s epochs, both from the cue onset. To obtain EEG and EMG artifact-free trials, we applied a semi-automatic procedure. Specifically, for the EEG trials we defined a voltage threshold (∓ 100 μV) and rejected all trials in which 5 channels exceeded the threshold, otherwise a spherical interpolation was performed to replace noisy channels and the trial was saved. As for the EMG trials, we applied a statistical criterion based on the comparison between the EMG characteristics of each trial and the median EMG characteristics of all trials (reference characteristic) [30] then the selected trials were visually inspected and validated.

As for the EXP group, the EEG time series recorded over different scalp positions from patients with right-sided lesions were flipped along the midsagittal plane so that the ipsilesional side was common to all patients. Similar procedure was also applied to EMG data in all the patients with left affected hand (right hemisphere lesion). Both flipping procedures thus ensured to label the left hemisphere and contralateral right hand as “affected” in all the patients, independently from their actual lesion side.

#### Corticomuscular coherence (CMC) pattern computation

The EEG signals were re-referenced according to the common average reference (CAR) to correctly localize CMC peaks over sensorimotor areas in agreement with physiology of movement, as it has been demonstrated elsewhere [31]. The EEG edge electrodes were excluded from the analysis due to the possible presence of artifacts related to facial movements, thus only 41 EEG electrodes were included in the analysis. EMG signals were rectified before entering the coherence computation.

Coherence is an indicator of the linear connection between two signals, and it is an extension of Pearson correlation coefficient in the frequency domain. It is defined as cross-spectra normalized by auto-spectra [32]:

1 $$Co{h}_{xy}\left({f}_{j}\right)=\frac{{\left|{S}_{xy}\left({f}_{j}\right)\right|}^{2}}{\left|{S}_{xx}\left({f}_{j}\right)\right|\cdot |{S}_{yy}\left({f}_{j}\right)|}$$

where ${S}_{xy}\left({f}_{j}\right)$ is the cross-spectrum of signal x and y, while ${S}_{xx}\left({f}_{j}\right)$ and ${S}_{yy}\left({f}_{j}\right)$ are the power spectral densities of x and y respectively at a given frequency ${f}_{j}$.

Typically to test whether CMC is significant, its values are compared to the chance level. However, in motor tasks it is mandatory to go beyond the null-case validation and thus, to assess the significance of the connections against rest condition to ensure that only the relationships related to the executed task are kept. Accordingly, we decided to use a non-normalized version of the CMC in (1) to prevent the detection of false positives in CMC when the muscle activation level is around 0, as expected in the rest time interval of our experiment [33].

The CMC was computed in a 2 s-window which were selected differently for Task and Rest condition. As for “task” trials, the interval of [5–7] s from cue onset was selected whereas we selected the first artifact-free interval of 2 s length in “rest” trials.

CMC values were estimated in the range [1–60] Hz for each participant, movement (ExtR/AH, ExtL/UH, GraspR/AH, or GraspL/UH) and interval of interest (Task, Rest). Two different procedures were followed for the CMC estimation: across-trials and single-trials for Group Analysis and Single Subject Analysis, respectively. As for the across-trials approach (periodogram window length of 1 s with 0% overlap) a single CMC pattern was estimated from all trials in the dataset of a single participant, in order to have an average of CMC pattern for each single participant to enter in the grand average (see Statistical analysis—GA patterns). As for the single-trial approach (periodogram window length of 0.250 s with 50% overlap), a CMC spectrum was estimated for each trial in the dataset, to obtain different observations of CMC patterns for each single participant. The CMC values were then extracted for the 3 considered frequency bands defined as alpha (8–12 Hz), beta (13–30 Hz) and gamma (31–60 Hz). For each of these bands, we identified the characteristic frequency as the frequency in which CMC showed the highest value for each pair of signals. The characteristic frequency was specific for each pair of signals, it was computed in the Task condition and used also for the Rest in order to compare patterns at the same frequency.

#### Analysis of CMC patterns properties by graph theory indices

CMC networks estimated at single-subject level were assessed against chance level and thus transformed into weighted CMC adjacency matrices. The single-subject CMC adjacency matrices were built as follows: for each EEG–EMG pair we applied an unpaired t-test between task and rest conditions on CMC values estimated by means the single-trial procedure. The significance level was set to 0.05. False Discovery Rate (FDR) was used to control family-wise error rate [34]. Such statistical comparison was used to assess CMC values obtained during movement execution/attempt against chance level using as null-case statistical threshold the corresponding CMC values in rest condition. The application of this test allowed to obtain for each subject and each movement a CMC adjacency matrix where null-values correspond to EEG–EMG connections not significantly different from rest while non-null values correspond to connections where CMC values were significantly higher during movement than rest condition. The comparison between task and rest conditions allowed also to reduce the presence of spurious connections in CMC networks due to volume conduction which is an intrinsic phenomenon of the EEG signals.

The Graph Theory was applied to the obtained CMC adjacent matrices to extract a set of ad hoc indices which synthetically described the main properties of the CMC patterns. This procedure aimed at reducing the CMC matrix complexity and thus allowing its interpretation.

Such computation was repeated for each subject, movement, and band.

Global network properties:

- *CMC Weight* is defined as the average of CMC values of the existing connections in the network. It is a measure of the strength of the EEG–EMG connections which is well-known to be reduced in stroke patients [13].
- *Network Density (ND)* computed as the total number of existing connections in the pattern normalized for the possible number of connections.

Network density was also calculated for each of the identified 4 sub-networks as follows (local networks properties):

- *Density (of) Contralateral Hemisphere (DCH)* calculated as the total number of existing connections that link the target muscle (FD in Grasp and ED in Ext) with EEG electrodes in contralateral hemisphere (normalized for the possible number of connections in this sub-network).
- *Density (of) Ipsilateral Hemisphere (DIH)* calculated as the total number of existing connections that link the target muscle (FD in Grasp and ED in Ext) with EEG electrodes in ipsilateral hemisphere (normalized for the possible number of connections in this sub-network).
- *Density (of) Involved Side (DIS)* calculated as the total number of existing connections entailing muscles in the side involved in a given motor task – target muscles (normalized for the possible number of connections in this sub-network).
- *Density (of) Uninvolved Side (DUS)* calculated as the total number of existing connections entailing muscles in the side which is not involved in a given motor task – non-target muscles (normalized for the possible number of connections in this sub-network).

To further investigate the selective engagement of muscles, we computed:

- *Muscle Degree (MD)* defined as the total number of connections that each muscle establishes with EEG channels normalized for the maximum number of possible connections involving it. This index allowed us to measure the involvement of each muscle in the pattern and to identify the muscles with a dominant role (higher degree) with respect to others. It was calculated for each of the recorded 16 muscles both during Ext and Grasp, and then a qualitative comparison was performed between the muscle degree values relative to the movement involved and uninvolved side.
- *Distal/Proximal Degree Ratio (DPDR)* was computed considering the degree of the muscles of the movement involved side, that were labeled as distal (FD and ED) and proximal (BIC, TRI, Ant_DELT, Lat_DELT, PEC, TRAP). It was defined as the ratio between the degree of distal muscles and the sum of degrees in distal and proximal muscles. DPDR value was set as equal to: 1 if the activation regarded only distal muscles; 0 for the activation of only proximal muscles; 0.5 in the case of both proximal and distal muscle activation with the same weight.

### Statistical analysis

#### Grand average (GA) CMC patterns

**Fig. 2:**
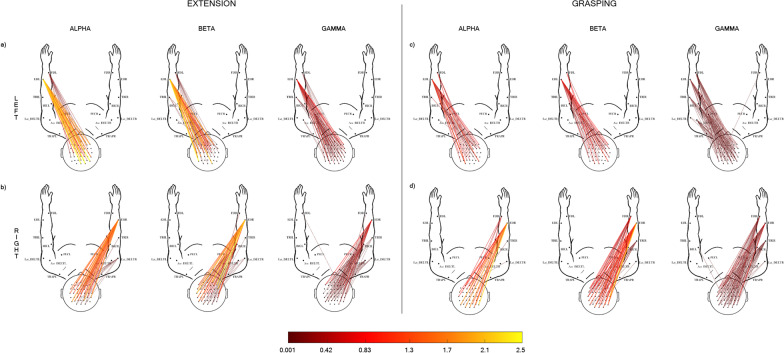
Grand average corticomuscular coherence patterns in CTRL group estimated for each of the 3 frequency bands, alpha (8–12 Hz), beta (13–30 Hz), gamma (31–60 Hz). Ext: CMC patterns obtained for the extension movement executed with left (**a**) and right (**b**) hand. Grasp: CMC patterns obtained for the grasping movement, executed with left (**c**) and right (panel **d**) hand. The 2D body model is seen from the above: scalp with nose pointing up the top and arms in front of the participant. Only statistically significant CMC values are represented (paired t-test between task and rest trials, α = 0.05 FDR correction). The color bar codes for the CMC average value (across participants, N = 12) in the task trial

**Fig. 3:**
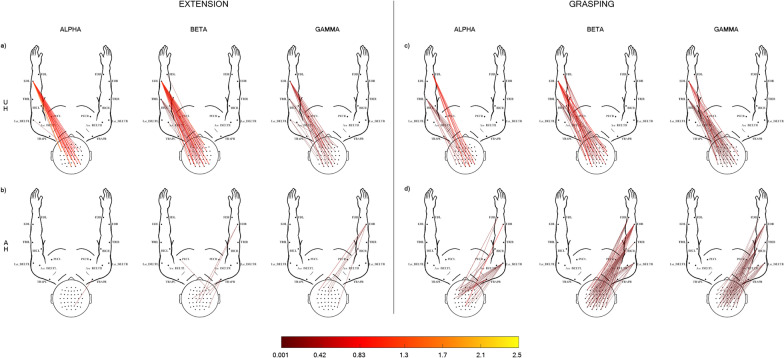
Grand average corticomuscular coherence patterns in EXP (stroke) group estimated for each of the 3 frequency bands, alpha (8–12 Hz), beta (13–30 Hz), gamma (31–60 Hz). Ext: CMC patterns obtained for the extension movement executed with UH (**a**) and AH (**b**). Grasp: CMC patterns obtained for the grasping movement, executed with UH (**c**) and AH (**d**). The 2D body model is seen from the above: scalp with nose pointing up the top and arms in front of the participant. Only statistically significant CMC values are represented (paired t-test between task and rest intervals, α = 0.05 FDR correction). The color bar codes for the CMC average value (across participants, N = 12) in the task trial

Each movement was described by a coherence pattern as a result of a GA analysis computed for the CMC values across participants (see Figs. 2 and 3). A paired sample t-test with the interval (Task vs Rest) as independent variable and the CMC values computed in the across-trials procedure as dependent variable was applied to each movement type, frequency band and channel pair. The significance level was set to 0.05. FDR was used to control family-wise error rate.

#### Between-groups differences in CMC pattern properties

A Kruskal–Wallis test was applied on each graph theory derived index considering as factor the three groups: CTRL—control group executing the task with the right hand; EXP_UH—stroke group executing the task with the unaffected hand; EXP_AH—stroke group executing the task with the affected hand. A Tukey’s post hoc test was applied to assess between groups differences. We selected the right hand for CTRL group since no significant differences were observed in the graph indices between left and right hand.

#### Correlation between brain network indices and functional/clinical scales

Brain network indices that significantly described the CMC patterns of stroke patients performing movements with the affected arm were correlated with the scores obtained from the following clinical scales: FMA total, FMA sub-scales and MMT. The Spearman’s correlation test was applied with the indices values as the dependent variable and the clinical scales’ scores as the independent variable.

## Results

### Participants

No significant between group (EXP and CTRL groups) differences were found in age (t-test p = 0.22) and number of subjects per gender (Chi-square test p = 0.08). All subjects in the CTRL group were right-handed according to the EHI. Ten patients in the EXP group were also right-handed while 2 were ambidextrous. Stroke severity was mild according to NIHSS which was lower or equal to 4 in all EXP participants [35]. Upper limb deficit as classified with FMA was mild to moderate, ranging from 23/66 to 63/66 [36]. See Table 1 for further details about participants.

**Table 1:** Demographic and clinical characteristics of the patients (means ∓ standard deviation)

GROUP	EXP (N = 12)	CTRL (N = 12)
AGE (YR)	52.5 (± 18.5)	43.6 (± 15.3)
HANDEDNESS	10RH + 2MH	12 RH
TIME FROM EVENT (MO)	5.5 (± 3.3)	–
TYPE (S/C)	6S + 6C	–
ETIOLOGY (I/H)	6I + 6H	–
SIDE OF LESION (R/L)	7L + 5R	–
FMA	49.4 (± 13)	–
NIHSS	2.42 (± 1.3)	–
MAS	0.9 (± 1.4)	–
MMT	20.3 (± 4.8)	–

C: Chronic; FMA: Fugl-Meyer Assessment scale, upper limb section, ranging from 0 (most affected) to 66 (least affected); H: Hemorrhagic; I: Ischemic; L: left; LH: left-handed; MH: mixed-handed; MAS: Modified Ashworth Scale; NIHSS: National Institute of Health Stroke Scale; R: right; RH: right-handed S: Subacute

### CMC grand average (GA) patterns

The Fig. 2 illustrates the GA CMC patterns obtained for the Ext (left panel) and Grasp (right panel) executed with left (panel a,c) and right (panel b, d) hand in CTRL group. As expected, these results confirmed our recent findings in [11]. In Ext condition (Fig. 2, Ext, panel a, b), we found the highest CMC values for connections involving mainly the target muscle (ED) and most of the bilateral sensorimotor EEG electrodes, in alpha and beta bands. In gamma band, CMC patterns were more diffuse involving almost all the muscle of the relative side and showed lower values of coherence with respect to those in the alpha and beta band. The Grasp condition (Fig. 2, Grasp, panel c, d) showed CMC values lower than those obtained in Ext. The target muscle FD was connected with almost all the electrodes over the bilateral sensorimotor areas in alpha band, whereas ED and proximal muscles were more involved in higher frequency bands (Beta, Gamma).

Different CMC patterns were observed in the stroke group (EXP) as illustrated in Fig. 3. First, the GA CMC patterns obtained in all experimental conditions showed a lower number of connections and lower CMC values with respect to the CTRL group (Fig. 3, Ext, Grasp), being the CMC lowest values observed in the AH condition (attempted movements; Fig. 3 b,d). The UH condition (Fig. 3a–c) revealed CMC patterns that mainly linked the bilateral sensorimotor areas with ED in Ext and FD in Grasp, respectively. Similar to what observed for the CTRL group, both tasks were characterized by a reduction of CMC values and a less specificity of the muscles involved in the task as the frequency increased. The GA CMC patterns were poor of significant connections when Ext and Grasp were executed with the affected hand (Fig. 3b–d). Very few connections were found between ED and bilateral sensorimotor areas during Ext. The CMC patterns were denser in Grasp condition with respect to Ext but they show less muscle selectivity, involving muscles other than the target ones even in alpha band.

### Analysis of CMC patterns by graph theory indices

**Fig. 4:**
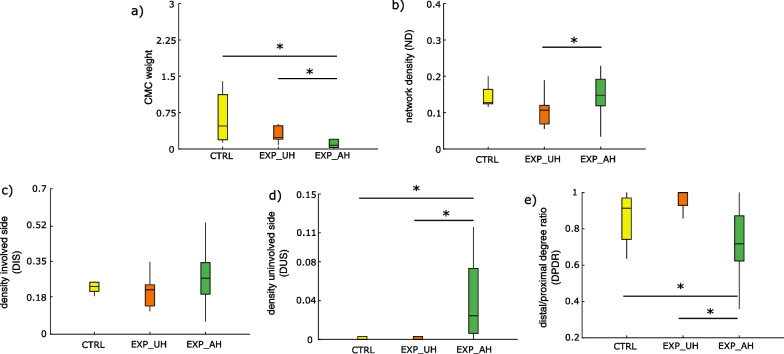
Box-plot diagrams reporting the distribution of graph theory derived indices characterizing CMC patterns in beta band during extension movement for the three different groups (CTRL, EXP-UH, EXP-AH). Each panel refers to as a specific index: **a** CMC weight, **b** network density, **c** density (of) involved side **d** density (of) uninvolved side, **e** degree ratio of distal/proximal muscle. The symbol * indicates a statistical difference as revealed by the post-hoc test

In Table 2 we reported the results of the between-group (CTRL, EXP-UH, EXP-AH) analysis on graph theory derived indices which characterized the CMC patterns in the different frequency bands and movements. The trends relative to these statistical differences are reported in Fig. 4 for beta band during Ext movement. A similar behavior was observed in the other two frequency bands (data not shown).

**Table 2:** Results of the kruskal–wallis test (p-values) obtained considering as dependent variables the different graph theory indices separately and as between factor the group (CTRL, EXP-UH, EXP-AH)

	Extension			Grasping		
	Alpha	Beta	Gamma	Alpha	Beta	Gamma
CMC weight	0.113	0.009*	0.014*	0.068	0.003*	0.006*
ND	0.033*	0.005*	0.001*	0.84	0.719	0.831
DCH	0.55	0.31	0.73	0.62	0.89	0.46
DIH	0.28	0.87	0.7	0.45	0.32	0.77
DIS	0.344	0.133	0.029*	0.934	0.776	0.384
DUS	0.012*	0.0001*	0.011*	0.125	0.337	0.299
DPDR	0.071*	0.004*	0.014*	0.503	0.551	0.982

Tests were repeated for each frequency band (alpha, beta, gamma) and each movement (Ext, Grasp). ND: network density; DIS: density involved side; DUS: density uninvolved side; DCH: density contralateral hemisphere; DIH: density ipsilateral hemisphere; DPDR: distal/proximal degree ratio

* indicates statistical significance

The CMC weight index estimated in beta and gamma bands was significantly different between the EXP and CTRL group in both Ext and Grasp conditions, showing lower weight when the EXP group performed Ext with AH with respect to UH and to the CTRL group (Fig. 4a).

A significant effect of the group factor was found for ND only for the Ext movement in all the frequency bands: higher connection density was observed for AH with respect to the UH in the EXP group (Fig. 4b). As for the sub-network density analysis, no between-group differences were found for densities in both ipsi- and contra-lateral hemispheres. On the other hand, significantly higher density of connections with muscles of the uninvolved side (DUS) were observed in all frequency bands (Table 2) when the movement was performed by the EXP group with AH (Fig. 4d) with respect to UH and to the CTRL group. A similar trend was observed for density in the involved side (Fig. 4c), reaching statistical significance only in gamma band (Table 2).

**Fig. 5:**
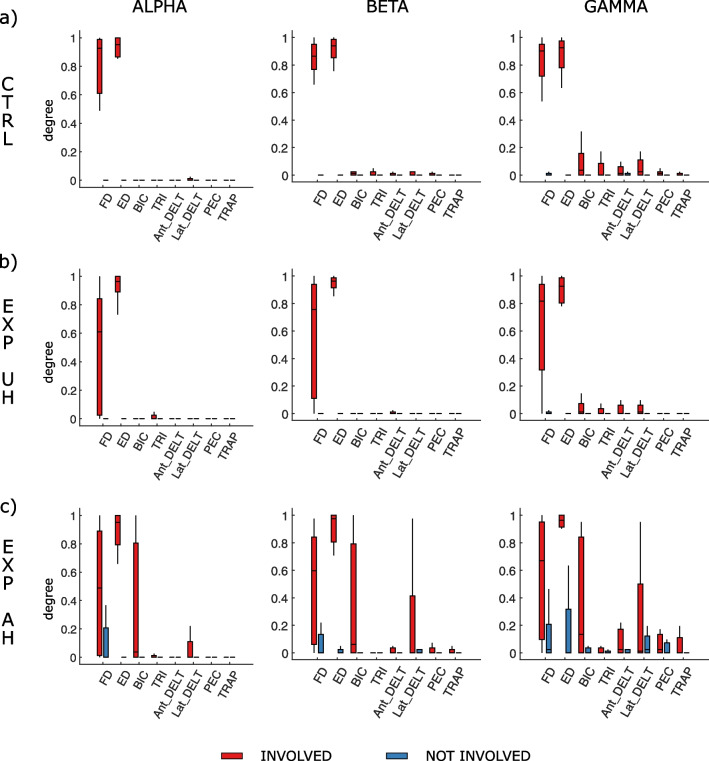
Degree distribution for each muscle in both involved and uninvolved sides for the 3 frequency bands during extension movement. Panels refer to as the CTRL group executing movement with right side (**a**) and the EXP group when the movement was executed with unaffected (UH, **b**) and affected (AH, **c**) side

Figure 5 illustrates the degree distribution for each of the 8 considered muscle in both arms for the 3 frequency bands during extension movement performed by CTRL and EXP group (similar results were observed for grasping). As for the CTRL group (Fig. 5a), maximum degree was observed for ED and FD in the involved side in all the frequency bands. The median was around 90% with a very short inter-quartile range, indicating a high reproducibility of this result across healthy participants. Degree close to zero was obtained for all the other muscles both in involved and uninvolved side in alpha and beta bands. Small degree (around 10%) was found only in gamma band for all the muscles in the involved side other than FD and ED reflecting the more diffuse CMC patterns at high frequencies.

As for the EXP group, a different behavior was found when the Ext was executed with unaffected (Fig. 5b) and affected (Fig. 5c) hand. Under the UH condition, the maximum degree was found for ED muscle (median around 95%) in almost all the patients (short inter-quartile range). Degree distribution for FD muscle showed a median around 60% with a high inter-quartile range, reflecting the variability among patients in the engagement of the FD during Ext task. The degree distribution of all the other muscles was similar to that described in CTRL group: zero degree of the uninvolved side in all the frequency bands; zero degree of all the muscles in the involved side other than ED and FD in alpha and beta bands; small degree (around 10%) for all the non-target muscles in the involved side in gamma band.

The observation of muscle degree distribution during movement attempt with AH showed a degree different from zero for most of the non-target muscles in the involved side with a high variance across the patients in all the frequency bands. Furthermore, a non-null degree was observed in muscles in the side not involved in the task (the unaffected side), especially of ED and FD in alpha and beta bands and of all other muscles in gamma band.

Significant between-group differences were observed for DPDR index for Ext movement in all frequency bands (Table 2). The EXP group showed a significantly lower DPDR when the movement was performed with AH with respect to UH and to the CTRL group (Fig. 4e). The DPDR is almost 1 in CTRL and in EXP-UH, reflecting the exclusive engagement of distal muscles in movement execution. Such ratio decreased to a median of 0.7 in the EXP group when the movement was attempted with AH, revealing a contribution of proximal muscles.

### Correlation of CMC patterns properties with clinical scales

Table 3 reports the results of the correlation analysis conducted between graph theory derived indices which significantly characterized the CMC patterns in Ext and Grasp movements, and the clinical scale scores describing upper limb motor function and strength (FMA total and subsections and MMT). Positive correlation was found between CMC weight and FMA-”Hand” subsection scores for Ext and between CMC weight and MMT for Grasp, in all the frequency bands. The DPDR index positively correlated with MMT for Grasp only in beta band. Negative correlation was observed between DIS index and FMA-”Hand” scores in alpha and beta bands for Ext and in gamma band for Grasp.

**Table 3:** Results of the correlation between the scores obtained for the two clinical scales, FMA and MMT, and each of the graph theory derived indices which significantly characterized the CMC patterns in stroke patients during AH condition

	FMA Hand						MMT					
	Extension			Grasping			Extension			Grasping		
	Alpha	Beta	Gamma	Alpha	Beta	Gamma	Alpha	Beta	Gamma	Alpha	Beta	Gamma
CMC weight	0.75*	0.72*	0.59*	0.36	0.46	0.44	0.57	0.53	0.49	0.62*	0.62*	0.58*
ND	− 0.55	− 0.55	− 0.52	− 0.54	− 0.53	− 0.58*	− 0.25	− 0.25	− 0.23	− 0.28	− 0.27	− 0.33
DIS	− 0.59*	− 0.59*	− 0.41	− 0.52	− 0.5	− 0.59*	− 0.24	− 0.28	− 0.12	− 0.27	− 0.26	− 0.31
DUS	− 0.53	− 0.3	− 0.44	− 0.35	− 0.2	− 0.17	− 0.3	− 0.12	− 0.3	− 0.06	− 0.002	0.08
DPDR	0.48	0.14	0.16	0.55	0.39	0.55	0.2	− 0.15	− 0.18	0.46	0.59*	0.48

The analysis was repeated for each frequency band (alpha, beta and gamma) and each movement (Ext, Grasp). ND: network density; DIS: density involved side; DUS: density uninvolved side; DPDR: distal/proximal degree ratio

* indicates statistical significance

## Discussion

The main objective of this study was to identify corticomuscular network properties which would describe the upper limb motor impairment in stroke patients, to ultimately guide the design of a novel hybrid BCI for motor recovery. To this aim, we analyzed and compared the CMC networks related to simple hand movements attempted with the affected hand and executed with the unaffected hand in stroke patients and those obtained from a sample of age-matched healthy participants performing the same movements with right and left hand.

As for healthy participants, our results retrace those recently obtained by our group [11]. We confirmed that CMC patterns observed during simple hand movements (Fig. 2) are widely distributed over the sensorimotor scalp areas, muscle involvement is more selective to the target muscle during extension than grasping, and less specific in higher bands for both movements. Furthermore, CMC values are lower for the grasping movement with respect to extension.

Grand average patterns obtained from stroke patients (Fig. 3) show much less connections with lower CMC values, both in the UH and AH conditions, probably due to a higher inter-subject variability as well as to the expected reduction in CMC weight (Table 2). Indeed CMC weight had significantly lower values in patients for both movements, under UH and AH conditions in beta and gamma bands, already identified as most significant to highlight brain-muscle communication disorders [8].

As evident in Fig. 3, grand average patterns in patients during AH are almost devoid of connection, especially for extension movement. As mentioned, we impute this shortage of connections in the grand average pattern to a high inter-subject variability among patients, that was possibly higher in the extension task with respect to grasping (see Additional file 1 for single-subject CMC patterns). Indeed, it might be argued that the extension task resulted more challenging to our patients and thus lead to individually distinct compensation strategies. The pattern for grasping with AH is slightly richer, possibly because grasping holds a high behavioral and functional complexity and that our patients were all undergoing a standard rehabilitative program likely including upper limb functional exercises when the experiments were performed. Nevertheless, with respect to grasping patterns from healthy subjects, patients showed lower muscle specificity in all bands. This result is largely expected from a revision of CMC literature in stroke patients, showing involvement of proximal muscles to compensate for distal impairment [22] or higher contribution of antagonist muscles with respect to healthy subjects [14, 23]. More generally, alterations of muscular involvement in post-stroke patients have largely been described through the phenomena of motor-overflow, co-activation of agonists and antagonists, spasticity and appearance of mirror movements [8].

To characterize these alterations through CMC pattern evaluation in a quantifiable and objective manner, we defined indices derived from graph theory and applied those to single-subject networks.

Overall network density was higher in the patient group for the AH condition (Ext movement only), suggesting that a higher number of connections in the network is required to accomplish the task. In classical graph theory, indeed, an increase in overall network density is described as a deterioration of such an optimal criterion according to which physiological networks are organized (well-known as small-world networks) [37]. This increase in overall density could be ascribed to compensatory strategies which were more relevant in the extension task. To further interpret this result and thus, to characterize deviations from the physiological condition, we split the index considering four sub-networks relative to the hemispheres contra- or ipsi- lateral to the hand task, and to the muscles on the side involved and uninvolved (contralateral) in the task.

As for the distribution of connections on the scalp, no statistical differences were observed in indices describing scalp lateralization of CMC patterns. Such bilateral distribution of connections was already observed in healthy subjects and discussed in our previous work [11]. The current findings on patients demonstrate that the presence of a unilateral stroke lesion does not affect this pattern distribution that remains balanced between the ipsilateral and contralateral hemisphere during movement with the healthy or paretic hand. This is not entirely expected according to the widely described interhemispheric unbalance of electrical activity after stroke [38]. However, our patients were all in subacute to chronic phase, with low level of impairment and undergoing a standard rehabilitative treatment when the experiments were performed. A lack of interhemispheric unbalance has already been associated with good recovery [39], thus it could be that more severe patients recorded closely to the stroke event might still show the differences in CMC pattern distribution between the affected and unaffected hemisphere that were not seen in our sample.

As for sub-networks related to muscles on the involved and uninvolved task side, while density values were higher in both the involved and uninvolved side (Fig. 4 panels c and d), the uninvolved side density only was significantly higher in patients for AH condition, demonstrating an abnormal recruitment of healthy side muscles during the extension task with the paretic side. Visible mirror movements were not present in our sample during AH tasks (except for two patients), however the occurrence of non-paretic upper limb movements during paretic motor attempts in stroke is largely described [7, 19]. Thus, we speculate that our analysis on CMC network properties might reveal subclinical alterations.

Muscle degree, i.e. the number of connections involving each recorded muscle was employed in order to quantify muscle specificity for each task. As expected, the target muscle of the involved side holds the highest degree in both groups and conditions (in Fig. 5, red ED bars). However, in the CTRL group and only in UH condition for EXP group all other muscles have very low degrees (except for low values appearing mainly in gamma band), while in the AH conditions several muscles are represented from the involved and contralateral side. Among those, the highest values are observed in the ipsilateral BIC muscle. The bicep is crucial for post stroke upper limb flexion spasticity [40] as testified by clinical studies [41, 42]. Despite the low or absent clinical spasticity in our patients (as assessed by MAS) we argue that this finding may represent a subclinical substrate for elbow flexion spasticity; future studies involving stroke patients showing higher level of spasticity are needed to definitely corroborate this argumentation.

As for the distal/proximal degree ratio, our results show that during AH in the EXP group, proximal muscles were involved confirming a compensatory proximal activity during hand motor tasks in paretic patients [14].

Altogether these findings confirm that CMC is a promising metric to analyze post-stroke changes in upper limb motor activity as it allows us to quantify commonly reported alterations (co-activation of proximal and contralateral muscles as possible substrates for spasticity and for mirror movements). To further evaluate the solidity of such a method to describe post-stroke upper limb motor impairment, we tested correlation of CMC indices with clinical/functional scales of the upper limb. Significant results were found for the FMA “Hand” subscale (mainly for the extension movement) and for MMT (grasping only). In particular, CMC weight was lower in more impaired patients. Similarly, the distal/proximal muscle degree ratio was lower in more impaired patients, proving the higher need of proximal compensation. Conversely, density and involved side muscle density were negatively correlated, showing a network organization that was more similar to healthy subjects in less impaired patients (lower density as a possible indicator of a higher network efficiency). With the caution required by the relatively small sample in our study, these results could be interpreted taking into account the differences between the two clinical scales. Indeed, FMA is a fairly complex scale which entails several aspects such as reflex activity, different functional movements and synergies, coordination, and speed; on the other hand, MMT is merely a measure of residual strength in different upper limb segments. It might be speculated that grasping being less challenging for stroke patients as compared to extension could be responsive to a grosser evaluation such as MMT, while correlations with FMA are observed for extension task as the scale reflects motor functional improvement in a more complex fashion.

To our knowledge, the present work is among the first to analyze CMC in stroke patients in terms of a widely distributed network (ie. considering several EEG scalp positions and muscles) [14, 21–23], and the first to apply a graph theoretical approach to such networks. In a recent study [43], Xi et al. applied graph theory to CMC networks in healthy subjects. The present work moves a step forward by defining specific indices apt to describe post-stroke movement alterations in a quantifiable and objective manner.

The ultimate goal of our investigation is to drive the design and implementation of a novel hybrid BCI system which will reinforce only those CMC network features that most resemble normal activation and thus subside favorable motor outcome. The findings of the present work indeed confirmed that the reinforcement of CMC throughout a BCI paradigm is desirable, as a reduction of its weight is correlated with upper limb motor impairment. Moreover, we identified CMC features that describe derangements from physiological motor system activation which will be discouraged along the BCI training protocol to counteract maladaptive changes. Preliminary studies are being carried out in our laboratory to translate CMC estimation in an online paradigm to optimize the timing of feedback delivery [44].

A major limitation of the present study is that the small number of patients included resulted in a consequent low variability in the degree of impairment. Indeed, most enrolled patients were mildly impaired and with little or no spasticity. This was mainly due to the complex experimental setup and relatively long experiment that could result too tiring (if at all doable) for more severe patients. Future steps will require an optimization of the setup and experimental protocol (even according to the results presented here) to be able to include more patients with different levels of impairment.

## Conclusions

In this paper, we showed that analysis of high-density CMC networks by means of graph theory indices can describe motor abnormalities in stroke patients during simple hand movements, which are the most commonly employed motor tasks in rehabilitative BCI paradigms. Our results will drive the implementation of a novel hybrid BCI system able to reinforce those CMC network features that most resemble normal activation and thus, subside favorable motor outcome. Indeed, correlations of graph theory indices with upper limb motor impairment support their use in wider clinical and rehabilitative applications. As an example, correlations between CMC network properties and clinical scales are promising for the application of such measurements as rehabilitation outcome metrics, in line with the constant need for evidence-based and personalized rehabilitation approaches [45, 46].

## Supplementary Information

**Additional file 1. **Reports the single-subject corticomuscular coherence patterns in EXP (stroke) group estimated for the extension and grasping movements attempted with the affected hand (AH).

## Data Availability

The datasets used and analysed during the current study are available from the corresponding author on reasonable request.

## Abbreviations

AH: Affected hand
Ant_DELT: Anterior deltoid
BIC: Biceps brachii
BCI: Brain-Computer Interface
CAR: Common Average Reference
CTRL: Control group
CMC: Cortico-muscular coherence
DCH: Density Contralateral Hemisphere
DIH: Density Ipsilateral Hemisphere
DIS: Density involved side
DUS: Density Uninvolved Side
DPDR: Distal/Proximal Degree Ratio
EHI: Edinburgh Handedness Inventory
ECG: Electrocardiography
EEG: Electroencephalography
EMG: Electromyography
EXP: Experimental group
Ext: Extension
ED: Extensor digitorum
FDR: False Discovery Rate
FD: Flexor digitorum superficialis
FMA: Fugl-Meyer Assessment
GA: Grand Average
Grasp: Grasping
L: Left
Lat_DELT: Lateral deltoid
TRI: Lateral head of the triceps muscle
MMT: Manual Muscle Test
MVC: Maximum Voluntary Contraction
MAS: Modified Ashworth Scale
MD: Muscle Degree
NIHSS: National Institute of Health Stroke Scale
ND: Network Density
PEC: Pectoralis major
R: Right
UH: Unaffected hand
TRAP: Upper trapezius
