# Supplementary material for: Exploring high-density corticomuscular networks after stroke to enable a hybrid Brain-Computer Interface for hand motor rehabilitation
Source: J Neuroeng Rehabil. 2023 Jan 14;20:5. doi: 10.1186/s12984-023-01127-6 (PMC9840279; doi:10.1186/s12984-023-01127-6)
Supplement: Supplementary file 1 — Additional file 1. Reports the single-subject corticomuscular coherence patterns in EXP (stroke) group estimated for the extension and grasping movements attempted with the affected hand (AH). [file 12984_2023_1127_MOESM1_ESM.docx]

# Exploring high-density corticomuscular networks after stroke to enable a hybrid Brain-Computer Interface for hand motor rehabilitation

Floriana Pichiorri^1*^, Jlenia Toppi^1,2*^, Valeria de Seta^1,2^, Emma Colamarino^1,2^, Marcella Masciullo^3^, Federica Tamburella^4^, Matteo Lorusso^4^, Febo Cincotti^1,2^, Donatella Mattia^1^

*^1^Neuroelectrical Imaging and Brain Computer Interface Lab, IRCCS Fondazione Santa Lucia, Rome, Italy, ^2^Dept. Of Computer, Control and Management Engineering, Sapienza University of Rome, Rome, Italy, ^3^Neurology and Neurovascular Treatment Unit, Belcolle Hospital, Viterbo, Italy, ^4^Laboratory of Robotic Neurorehabilitation (NeuroRobot Lab), Neurorehabilitation 1 Department, IRCCS Fondazione Santa Lucia, Rome, Italy*

# Additional File 1


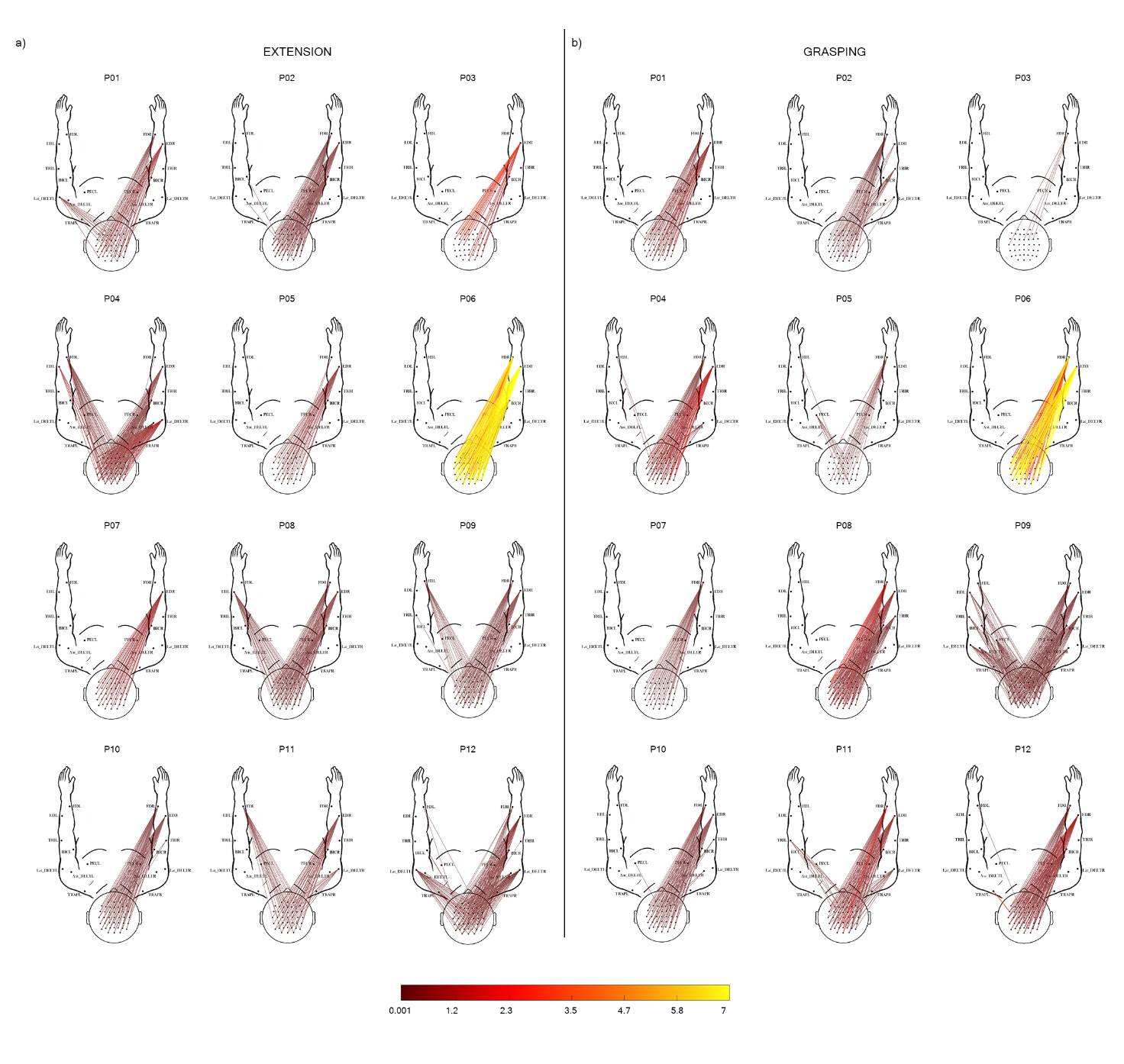


Figure S1. Single-subject corticomuscular coherence patterns in EXP (stroke) group estimated for alpha band (8–12 Hz) during extension (panel a) and grasping (panel b) movement attempted with the affected hand (AH). The 2D body model is seen from the above: scalp with nose pointing up the top and arms in front of the participant. Only statistically significant CMC values are represented (unpaired t-test between task and rest intervals, α =0.05 FDR correction). The color bar codes for the CMC average value across trials for each stroke participant (N participants = 12) in the task trial.


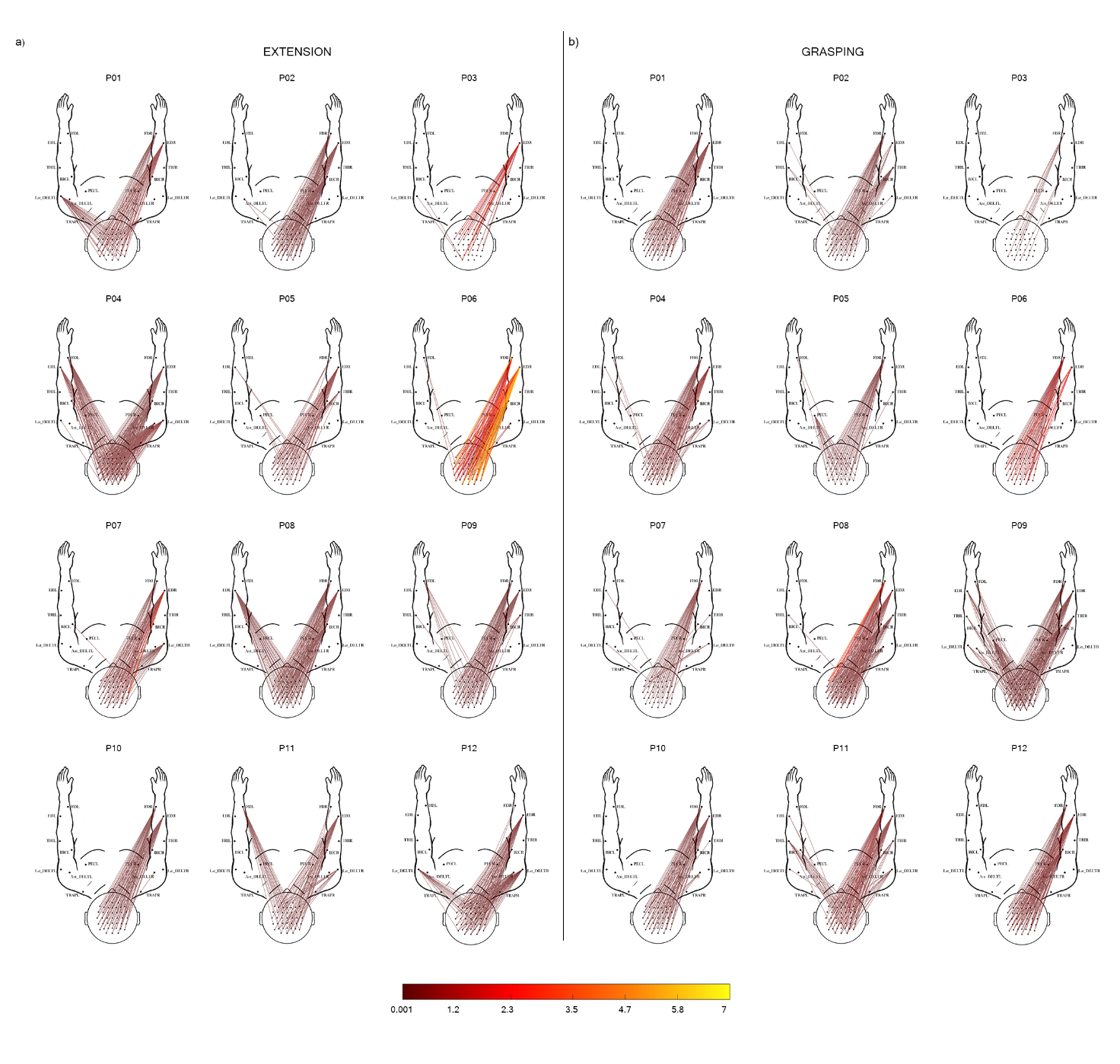


Figure S2. Single-subject corticomuscular coherence patterns in EXP (stroke) group estimated for beta band (13–30 Hz) during extension (panel a) and grasping (panel b) movement attempted with the affected hand (AH). The 2D body model is seen from the above: scalp with nose pointing up the top and arms in front of the participant. Only statistically significant CMC values are represented (unpaired t-test between task and rest intervals, α =0.05 FDR correction). The color bar codes for the CMC average value across trials for each stroke participant (N participants = 12) in the task trial.


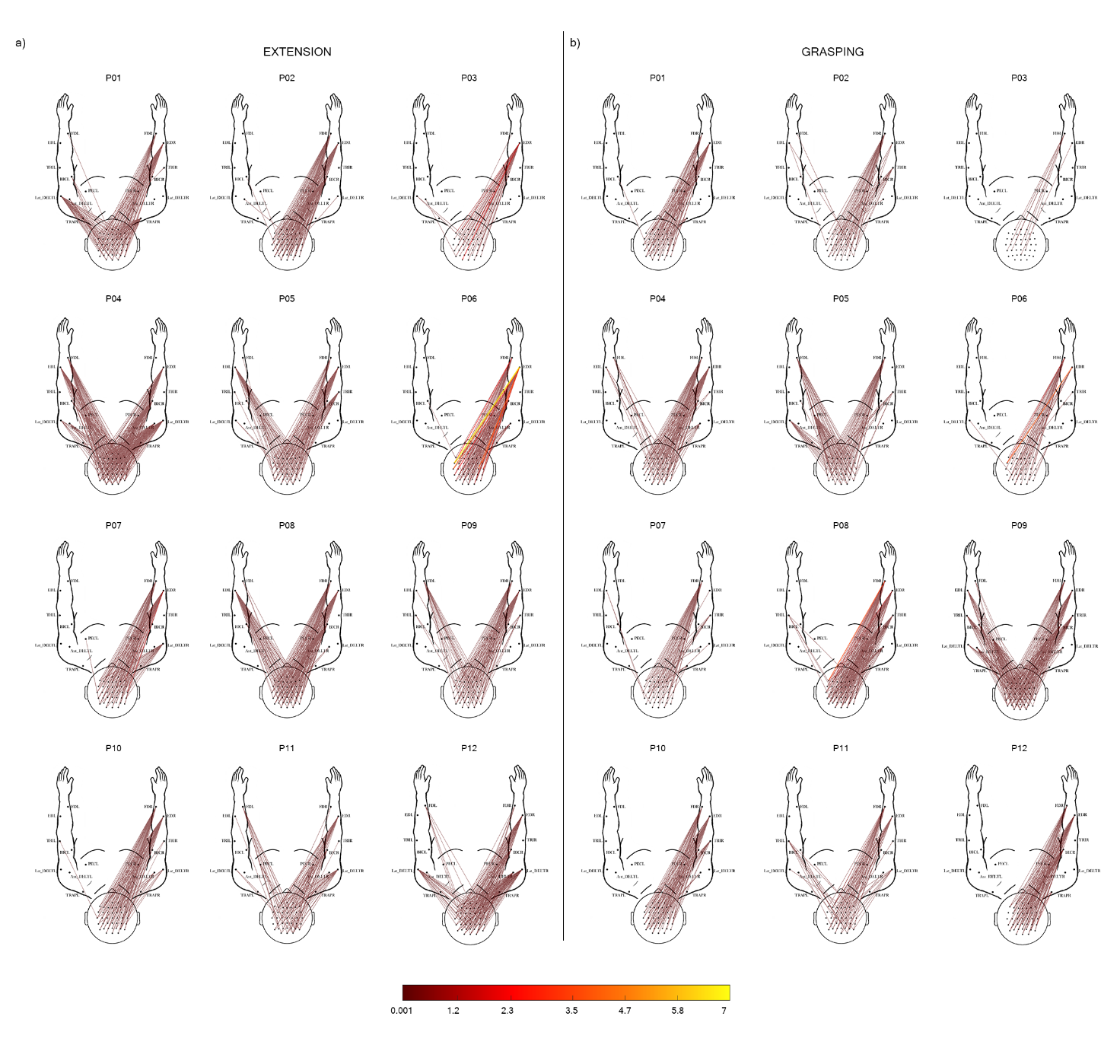


Figure S3. Single-subject corticomuscular coherence patterns in EXP (stroke) group estimated for gamma band (31–60 Hz) during extension (panel a) and grasping (panel b) movement attempted with the affected hand (AH). The 2D body model is seen from the above: scalp with nose pointing up the top and arms in front of the participant. Only statistically significant CMC values are represented (unpaired t-test between task and rest intervals, α =0.05 FDR correction). The color bar codes for the CMC average value across trials for each stroke participant (N participants = 12) in the task trial.
